# Editorial: Chromosome structural variants: Epidemiology, identification and contribution to human diseases

**DOI:** 10.3389/fgene.2022.1022918

**Published:** 2022-09-09

**Authors:** Zirui Dong, Dezso David, Claudia Gonzaga-Jauregui, Cynthia C. Morton, Cinthya J. Zepeda-Mendoza

**Affiliations:** ^1^ Department of Obstetrics and Gynaecology, The Chinese University of Hong Kong, Hong Kong SAR, China; ^2^ Shenzhen Research Institute, The Chinese University of Hong Kong, Shenzhen, China; ^3^ Hong Kong Hub of Paediatric Excellence, The Chinese University of Hong Kong, Hong Kong SAR, China; ^4^ Department of Obstetrics and Gynaecology, The Fertility Preservation Research Center, The Chinese University of Hong Kong, Hong Kong SAR, China; ^5^ Department of Human Genetics, National Health Institute Doutor Ricardo Jorge, Lisbon, Portugal; ^6^ International Laboratory for Human Genome Research, Laboratorio Internacional de Investigación sobre el Genoma Humano, Universidad Nacional Autónoma de México, Juriquilla, Querétaro, Mexico; ^7^ Department of Obstetrics and Gynecology, Brigham and Women’s Hospital, Boston, MA, United States; ^8^ Department of Pathology, Brigham and Women’s Hospital, Boston, MA, United States; ^9^ Harvard Medical School, Boston, MA, United States; ^10^ Broad Institute of MIT and Harvard, Cambridge, MA, United States; ^11^ Manchester Centre for Audiology and Deafness, School of Health Sciences, University of Manchester, Manchester, United Kingdom; ^12^ Division of Laboratory Genetics and Genomics, Department of Laboratory Medicine and Pathology, Mayo Clinic, Rochester, MN, United States

**Keywords:** structural variant (SV), methodologies & tools, SV spectrum, sequence complexity, annotation and prediction, genomic variation, genomic rearrangements, SV mechanisms

Human chromosome structural variants (SVs) are balanced/unbalanced genomic abnormalities that include translocation, inversion, insertion, and deletion/duplication (also known as copy-number variants, CNVs) events with a size of >50 bp. Currently, the capability of genome sequencing in the research and clinical fields has increased our capacity to detect cryptic SVs and further delineate the complexity of karyotypically/microarray detectable SVs. This has increased our knowledge of pathogenicity mechanisms by considering dysregulation of gene expression through position effects and complex interactions between gene dosage and mutational burden. However, much of the contribution of SVs to human disease is left to explore, as the incidence of SVs is still underestimated owing to limitations of current sequencing technologies and analytical pipelines, and few studies have comprehensively integrated SV information with single nucleotide variants in congenital diseases. Rigorous investigation of SV pathogenicity is warranted for clinical applications.

The Research Topic in this issue is divided into three main sections: three articles demonstrate methodologies in SV identification and pathogenicity annotation; five papers discuss the spectrum of SVs in individuals with different indications; and two reports characterize sequence complexity of SVs.

## Methodologies in SV identification and pathogenicity annotation

1) Chen et al. describe an optimized analytical approach in non-invasive prenatal testing (NIPT) by combining Z-score with maternal CNV analysis. In routine NIPT analysis, the calculation of Z-score approach is commonly used for determining whether the fetus has a numerical disorder. However, among those cases with outliers of Z-scores (such as Z > 3 or Z < −3), the presence of maternal CNVs should be considered. After verification with diagnostic prenatal diagnosis, the authors suggest conducting Z-score analysis together with identification of maternal CNVs to reduce significantly the false positive calling rate. 2) Guo et al. propose a new method, namely stLFRsv (single-tube Long Fragment Read), for identifying SVs with the use of co-barcoded reads. Co-barcoded reads originating from long DNA fragments provide long-range genomic information with single-base level accuracy superior to a long-read sequencing approach; however, no analytical method for SV analysis is available. The authors show a higher accuracy of SV detection utilizing co-barcoded reads through identification of abnormal large gaps between co-barcoded reads to detect potential breakpoints for reconstructing complex SVs and further filtering via haplotype phasing analysis. 3) Fino et al. present a web-based application, SVInterpreter, for annotation of both balanced and unbalanced SVs using topologically associated domains (TADs) as genome units. With the advancement of detection methods, a significantly increasing number of SVs are detected in both patients and presumably healthy individuals, and most of these SVs are interpreted as variants of uncertain significance (VUS) due to limited knowledge of their pathogenicity. By incorporating gene-associated data (as function and dosage sensitivity), phenotype similarity scores, and CNV scoring metrics, the authors demonstrate that SVInterpreter identifies the possible disease-causing candidate (such as contributed by potential position effect events) and decreases interpretations of VUS by 40%.

## SV spectrum in individuals with different indications

SVs are known to contribute to genomic diversity and diseases in individuals in different developmental stages: early miscarriage, prenatal, postnatal, and adult as well as serve as markers for somatic mutagenesis after exposure to a toxic environment. 1) Gu et al. show an uneven distribution of CNVs (<3 Mb in size) in euploid products of conception (POCs) with a higher density seen in the pericentromeric and subtelomeric regions, and the genes involved are significantly enriched in biological processes and pathways important to embryonic/fetal development. 2) Chau et al. examine the landscape of rare CNVs with parental inheritance assignment in trio-based prenatal diagnosis and demonstrate among 31 pathogenic/likely pathogenic CNVs identified, over 25% are small or mosaic CNVs unlikely to be detected by routine methods. 3) Hu et al. recruit seven Chinese prenatal cases with 21q21.1–q21.2 aberrations with comprehensive pedigrees, and demonstrate a benign clinical interpretation for pathogenic assessment of 21q21.1–q21.2 duplication and deletion, which were considered VUS or likely pathogenic in previous studies. 4) Lee et al. applied an in-house bioinformatics pipeline to 1,737 cases with Alzheimer Disease (AD) and 2,063 cognitively normal controls; burden tests show that Non-Hispanic White cases on average have 16 more duplications than controls, and Hispanic cases have larger deletions than controls. 5) Meléndez-Flórez et al. show that farmers exposed to pesticides had significantly increased frequencies of chromosomal alterations/variants, instability and clonal heterogeneity when compared with controls, which might contribute to an increased risk of developing diseases.

## Sequence complexity of SVs

The advancement of different methodologies helps in the delineation of sequence complexity and composition of SVs, which potentially contribute to diseases through different mechanisms such as gene disruption or dysregulation. 1) Cao et al. applied mate-pair low-pass genome sequencing in cases with developmental disorders and/or intellectual disabilities and demonstrate that a large proportion of duplications previously classified as VUS are forward tandem duplications without contributing to diseases due to gene disruption. 2) Grochowski et al. describe a 5-year-old female presenting with a constellation of clinical features consistent with a clinical diagnosis of Coffin–Siris syndrome 1 (CSS1), which is contributed by *ARID1B* gene disruption resulting from a *de novo* pericentric and multiple paracentric inversions from a chromoanagenesis-like event.

Overall, studies from this Research Topic not only provide state-of-the-art methods for identification, delineation, and pathogenicity annotation of SVs, but also elucidate the incidence, spectrum, sequence complexity and potential contribution to human diseases.

